# Neurootologische Manifestationen bei Morbus Fabry – eine retrospektive Analyse

**DOI:** 10.1007/s00106-023-01360-4

**Published:** 2023-09-25

**Authors:** Katharina Storck, Anna Stenzl, Claudia Regenbogen, Benedikt Hofauer, Andreas Knopf

**Affiliations:** 1 Klinik und Poliklinik für Hals-Nasen-Ohrenheilkunde, Klinikum rechts der Isar, TU München, München, Deutschland; 2 Innere Medizin II, Nephrologie, Klinikum rechts der Isar, TU München, München, Deutschland; 3 Klinik und Poliklinik für Hals-Nasen-Ohrenheilkunde, Universität Freiburg, Freiburg, Deutschland; 4 Klinik und Poliklinik für Hals-Nasen-Ohrenheilkunde, Klinikum rechts der Isar, Technische Universität München, Ismaningerstr. 22, 81675 München, Deutschland

**Keywords:** Hörminderung, Morbus Fabry, Neurootologische Defizite, X‑chromosomal lysosomale Speicherkrankheit, α‑Galaktosidase, Hearing loss, Fabry disease, Neurotological deficit, X‑linked lysosomal storage diseases, α‑galactosidase

## Abstract

**Hintergrund:**

Morbus Fabry (FD) gehört zu den X‑chromosomal lysosomalen Speicherkrankheiten, die alle Organe betreffen können. Ihnen ist eine spezifische lysosomalen Funktionsstörung gemeinsam, wodurch es – anstatt zum Abbau von Metaboliten – zur Substratanreicherung in Lysosomen kommt. Aufgrund des Mangels/Fehlens von α‑Galaktosidase werden Globotriaosylceramide (Gb3) in Lysosomen der Organe abgelagert. Neben Akroparästhesien, Angiokeratomen, autonomen Dysfunktionen, Vortex-Keratopathien, ischämischen zerebralen oder kardialen Komplikationen und einer chronischen Niereninsuffizienz kommen auch vestibulocochleäre Funktionsstörungen mit einem plötzlichen oder fortschreitenden asymmetrischem Hörverlust, Tinnitus und Schwindel vor.

**Patienten und Methoden:**

In dieser retrospektiven Studie erfolgte die Auswertung von 33 Patienten (m = 16 und w = 17) mit FD. Alle Patienten wurden uns im Rahmen einer Routineuntersuchung vom spezialisierten Zentrum für lysosomale Speicherkrankheiten der Nephrologie im Haus vorgestellt. Diese Vorstellung erfolgt als Screening-Untersuchung unabhängig von neurootologischen Symptomen.

**Ergebnisse:**

Das Durchschnittsalter bei Diagnose betrug 34,76 (±11,55) Jahre. Die erstmalige Vorstellung in unserer HNO-Abteilung erfolgte mit 40,45 (±11,71) Jahren. Wir konnten eine signifikante Korrelation zwischen neurologischen Symptomen und einer Hörminderung (*p* = 0,001) sowie zwischen Herzmanifestationen und einer Hörminderung (*p* = 0,024) nachweisen.

**Schlussfolgerung:**

Schwerhörigkeit ist ein mögliches Symptom bei Morbus Fabry und nicht nur auf den klassischen männlichen Phänotyp beschränkt. Bei nachgewiesenen Korrelationen mit neurologischen und kardiologischen Manifestationen der Krankheit sollte eine routinemäßige HNO-Screening-Untersuchung erfolgen, um neurootologische Defizite zu erkennen und behandeln zu können. Ebenso sollte vor allem bei jüngeren Patienten mit einem plötzlichen ein- oder beidseitigen Hörverlust und einer familiären Häufung FD als Differenzialdiagnose in Betracht gezogen und getestet werden.

## Abstract (en)

**Background:**

Fabry disease (FD) is one of the X‑linked lysosomal storage diseases that can affect any organ. They have a specific lysosomal dysfunction in common, which results in substrate accumulation in lysosomes instead of metabolite degradation. Due to the deficiency/absence of α‑galactosidase, globotriaosylceramides (Gb3) are deposited in lysosomes of the organs. In addition to acroparesthesia, angiokeratomas, autonomic dysfunction, vortex keratopathies, ischemic cerebral or cardiac complications and chronic renal failure, also vestibulocochlear dysfunctions with sudden or progressive asymmetric hearing loss, tinnitus and vertigo may be observed.

**Patients and methods:**

In this retrospective study, 33 patients (men = 16 and women = 17) with FD were evaluated. All patients presented to us in interdisciplinary cooperation as part of routine examinations by the specialized center for lysosomal storage diseases of the in-house department of nephrology. This presentation is carried out as a screening examination independent of neuro-otological symptoms.

**Results:**

The mean age at diagnosis was 34.76 (±11.55) years. The first presentation in our ENT department was at 40.45 (±11.71) years. We were able to demonstrate a significant correlation between neurological symptoms or apoplexy and hearing loss (*p* = 0.001) and between cardiac manifestations and hearing loss (*p* = 0.024).

**Conclusion:**

Hearing loss is a potential symptom of Fabry disease and is not limited to the classic male phenotype. Due to possible positive correlations with neurological and cardiological manifestations of the disease, routine ENT screening examinations should be carried out to be able to identify and treat neuro-otological deficits at an early stage. In addition, FD should also be considered and tested as a differential diagnosis, especially in younger patients with sudden unilateral or bilateral hearing loss and a family history.

Morbus Fabry („Fabry disease“, FD) gehört zu den X‑chromosomal lysosomalen Speicherkrankheiten, mit multilokulärem Organbefall und progredient fortschreitendem Verlauf [5]. Diesen Speicherkrankheiten ist eine spezifische lysosomalen Funktionsstörung gemeinsam, wodurch es anstatt zum Abbau von Metaboliten zu deren Substratanreicherung in Lysosomen kommt. Aufgrund des Mangels oder Fehlens von α‑Galaktosidase werden Globotriaosylceramide (Gb3) in Lysosomen verschiedener Organe abgelagert.

FD zeichnet sich durch den Mangel des Enzyms α‑Galaktosidase A (α-Gal) aus. Das kodierende Gen befindet sich auf dem langen Arm des X‑Chromosoms (Xq22.1). Beide Geschlechter können durch eine reduzierte Lebenserwartung betroffen sein, wobei hemizygote Männer stärker betroffen sind als heterozygote Frauen [19].

Die Inzidenz wird mit 1:40,000 bis 1:117,000 angegeben [21], neuere Studien gehen jedoch von einem höheren Vorkommen aus, welches vor allem auf pathogenetische Mutationen mit demografischen und ethnischen Auswirkungen zurückzuführen ist [13, 25].

Aufgrund des Mangels an α‑Gal kommt es zu einer Ablagerung von Glykosphingolipiden, hauptsächlich Globotriaosylceramid. Dies führt zu einer selektiven Beeinträchtigung der renalen, glomerulären und tubulären Epithelzellen, der Herzmuskelzellen und der Herzklappenfibrozyten, der Spinalganglien des autonomen Nervensystems sowie der endothelialen glatten Muskelzellen der Blutgefäße [5].

In frühen Stadien klagen die Patienten über Akroparästhesien der Extremitäten, Angiokeratome, autonome Dysfunktion und Vortexkeratopathien bereits im Kindes- oder Jugendalter [12]. In späteren Stadien führt die fortschreitende Glykolipidablagerung in den Endothel- und glatten Muskelzellen der Mikrovaskulatur zu einer Verengung und Thrombose kleiner Arterien. Dies führt zu ischämischen Komplikationen mit Beteiligung des Gehirns und des Herzens in Verbindung mit einer chronischen Niereninsuffizienz und häufig frühem Tod [3, 9].

Ein weiteres häufiges Symptom bei Fabry-Patienten sind Funktionsstörungen des vestibulocochleären Systems, die zu plötzlichem oder fortschreitendem asymmetrischem Hörverlust sowie zu vestibulären Funktionsstörungen mit Schwindel führen können [8].

Der genaue Mechanismus der Innenohrerkrankung ist noch nicht bekannt und wird kontrovers diskutiert. Ein vaskulärer Mechanismus mit Gb3-Ablagerungen im zuführenden Gefäßendothel scheint bei cochleärer Beteiligung eine Rolle zu spielen, während eine vestibuläre Beteiligung eine direkte Verletzung zu sein scheint [7]. Eine histopathologische Studie von Schachern et al. zeigte in den Felsenbeinknochen von zwei männlichen Patienten keinen Hinweis auf eine Ablagerung von Glykosphingolipiden in der Cochlea. Die Autoren berichteten lediglich von hyperplastischer Schleimhaut und einem seropurulentem Erguss im Mittelohr sowie von einer Atrophie des Strial- und Spiralbands und dem Verlust der äußeren Haarzellen [23].

Neurootologische Defizite bei FD rücken vermehrt in den Fokus. So berichtet beispielsweise Yazdanfard et al. in einer groß angelegten audiologischen Studie bei FD-Patienten von einem häufigeren Hörverlust bei Männern in höheren Frequenzen, wobei ein niedrigerer Gb3-Plasmaspiegel hierbei mit einem besseren Hörvermögen korreliert [27]. In einer Studie von Eyermann et al. wurde von einem Hörverlust bei über der Hälfte der eingeschlossenen FD-Patienten (62,2 %) berichtet, welcher sich ebenfalls überwiegend im Hochtonbereich manifestierte [7]. Die meisten Studien bezogen sich vor allem auf männliche Patienten, da in der Vergangenheit angenommen wurde, dass heterozygote Frauen nur Genträger sind [4, 10, 11, 14, 19, 20]. Neuere Studien berichten auch von weiblichen Patienten mit unterschiedlichen HNO-Manifestationen, je nach X‑chromosomalem Inaktivierungsmuster [7, 27].

Die Daten bezüglich eines potenziellen Hörverlusts bei Fabry-Patienten sind sehr unterschiedlich. Germain et al. beschreiben einen fortschreitenden Hörverlust bei 22,7 % der Patienten, einen plötzlichen Hörsturz bei 31,8 % und Tinnitus bei 22,7 % der Patienten [9]. Keilmann et al. beschreiben Veränderungen im Audiogramm bei 78 % eines Kollektivs aus 98 Patienten, wobei 38 % unter einem Tinnitus litten [14]. Hajioff et al. beschreiben einen ein- oder beidseitigen Hörverlust bei 80 % von 15 Patienten [11]. Die Daten von Eyermann et al. weisen einen Hörverlust von 62,2 % mit einer vestibulären Beteiligung von 56 % auf [7]. Rodrigues et al. eruierte eine Gesamtprävalenz otologischer Beschwerden von 34,4 %, wobei Schwerhörigkeit als häufigstes Symptom (26,2 %) angegeben wird. Es folgen in absteigender Häufigkeit Tinnitus (23 %), Benommenheit (6,6 %) und Schwindel (4,1 %) [22].

Seit Anfang der 2000er-Jahre erweist sich der klinische Einsatz von Enzymersatztherapien mit Infusionen aus gereinigter α‑Galaktosidase A als sicher und biochemisch aktiv [6, 24].

Diese Studie soll das Vorhandensein eines potenziell relevanten Zusammenhangs zwischen dem Auftreten von Funktionsstörungen des vestibulocochleären Systems und systemischen Manifestationen evaluieren.

## Patienten und Methoden

Es erfolgte die retrospektive Auswertung von 33 Patienten mit gesichertem FD, die sich im Zeitraum von 2011–2021 in unserer Ambulanz vorstellten. Alle Patienten wurden in interdisziplinärer Zusammenarbeit mit dem spezialisierten Zentrum für lysosomale Speicherkrankheiten der Abteilung für Nephrologie im Rahmen von Routineuntersuchungen an uns überwiesen, unabhängig von möglichen HNO-Symptomen oder Komorbiditäten. Auf diese Weise kann ein Präselektionsbias ausgeschlossen werden. Diese Studie wurde von der Ethikkommission an der Technischen Universität München bewilligt (Bewilligungsnummer 37/22). Die Diagnose FD wurde bei allen Patienten auf Basis der klinischen Anamnese und des α‑GAL-Enzymmangels gestellt und war bereits gesichert durch eine dann-Testung und mittels eines α‑Galaktosidase-Essays.

Die medizinischen Unterlagen enthielten allgemeine Informationen über die Erstdiagnose von FD, die familiäre Häufung, die systemischen und HNO-spezifischen Manifestationen der Krankheit und Informationen über die Enzymersatztherapie. Die systemischen Manifestationen wurden bereits durch die Kollegen der entsprechenden Fachabteilungen diagnostiziert und waren in Dokumenten im SAP hinterlegt.

Die spezifischen HNO-Informationen umfassten eine Anamnese über Hörverlust, Tinnitus und Schwindel sowie eine vollständige HNO-Untersuchung mit Ohrmikroskopie, Rhinoskopie, Inspektion der Mundhöhle und des Kehlkopfs. Die Untersuchung umfasste auch die Erfassung eines Spontannystagmus, provozierten Nystagmus und vibrationsinduzierten Nystagmus mittels Frenzel-Brille. Die Probanden wurden auch auf Traumata, Operationen am Ohr und andere Kopf- und Hals-Erkrankungen wie Tumoren überprüft. Ausschlusskriterien beinhalteten Exposition gegenüber ototoxischen Substanzen (z. B. Aminoglykoside), familiäre Schwerhörigkeit, Lärmbelastung oder akustische Traumata. Keiner der eingeschlossenen Patienten wies eine positive Anamnese für eines der genannten Ausschlusskriterien auf.

### Neurootologische Untersuchungen

Alle audiologischen Untersuchungen wurden mit kalibrierten Instrumenten in einem schallgedämmten Raum (DIN EN ISO 8253) durchgeführt. Die audiologischen Untersuchungen beinhalteten ein Reintonaudiogramm für Luftleitung (0,25 bis 8 kHz) und Knochenleitung (0,25–6 kHz), durchgeführt mittels eines Audiometers (AT 1000, Fa. Auritec, Hamburg, Deutschland) in 5‑dB-Schritten. Zudem erfolgte eine Tympanometrie und Stapediusreflexe (GSI Grason-Stadler Solutions, Eden Prairie, MN, USA), die von erfahrenen Audiologen in einem ruhigen Raum durchgeführt wurden. Außerdem unterzogen sich 19 Patienten einer auditorischen Hirnstamm-Audiometrie („brainstem-evoked response audiometry“, BERA; Eclipse by Interacoustics, Diatec, Dortmund, Deutschland). Bei der Standard-BERA werden die AEP (akustisch evozierten Potenziale) als Antwort auf einen Click-Reiz registriert. Bei den genannten Patienten wurden frequenzspezifische Messungen in Form einer Tonschwellenaudiometrie durchgeführt.

Die vestibuläre Diagnostik beinhaltete eine kalorischer Spülung, einen Blickfolgetest und eine Drehstuhltestung, die per Videookulographie (VOG) aufgezeichnet wurde und deren Durchführung ebenfalls den internationalen Normen entsprach.

### Klinische Datenanalyse

Die Art der Schwerhörigkeit wurde als Schallempfindungsschwerhörigkeit, Schallleitungsschwerhörigkeit oder kombinierte Schwerhörigkeit klassifiziert, gemäß der European Working Group on Genetics of Hearing Impairment (https://cordis.europa.eu/project/id/BMH4960353). Schallempfindungsschwerhörigkeit wurde als ein durchschnittlicher Abstand der Luft-Knochen-Leitung von weniger als 15 dB für 0,5 und 1 kHz definiert. Patienten mit einer Schallleitungsschwerhörigkeit wurden ausgeschlossen.

### Statistische Auswertung

Mögliche Zusammenhänge zwischen dem Hörverlust und anderen klinischen Manifestation wurden in Zusammenarbeit mit einer externen Firma für medizinische Statistik anhand der Kaplan-Meier-Kurve, der Cox-Regression für die Vorwärtsselektion und des Log-Rang-Tests untersucht.

## Ergebnisse

Die Krankenakten von 33 Patienten (16 Männer, 17 Frauen) mit bestätigtem FD wurden einbezogen. Die Tab. 1 gibt einen Überblick über alle eingeschlossenen Patienten mit FD inklusive der Systemmanifestation und neurootologischen Symptome. Alle Patienten zeigten einen unauffälligen Otoskopiebefund. Das Durchschnittsalter bei der Diagnose betrug 34,76 (±11,55) Jahre. Das Durchschnittsalter bei der erstmaligen Vorstellung in der HNO-Abteilung betrug 40,45 (±11,71) Jahre. Von den Patienten (24/33) waren 72,7 % bereits unter Therapie mit einer Enzymsubstitution.

**Table 1:** 

Variablen	Anzahl *n*	Häufigkeit	Gültige Prozent	Mittelwert	STD (±)	MIN	MAX
*Geschlecht*							
Weiblich	33	17	51,5	–			
Männlich	33	16	48,5				
*Alter bei Erstdiagnose (Jahre)*	33	–		34,76	11,554	14	59
*Alter bei Vorstellung (Jahre)*	33	–		40,45	11,713	20	66
*Patienten unter 45 Jahren bei Erstvorstellung*	33	21	–	37,6	7,3	20	45
*Weiblich*	*17*	*–*					
Alter bei Erstdiagnose	–			37,06	9,490	23	59
Alter bei Vorstellung				41,53	10,955	27	66
*Männlich*	*16*	*–*					
Alter bei Erstdiagnose	–			32,31	13,280	14	58
Alter bei Vorstellung				39,31	12,726	20	58
*Gentest erfolgt*	33	31	93,9	–			
*Enzymersatztherapie*	33	24	72,7				
*Symptome*							
Akroparästhesien	33	20	60,6	–			
Angiokeratome	33	10	30,3				
Neurologisch/Apoplex	33	13	39,4				
Gastrointestinal	33	13	39,4				
Nephrologisch	33	11	33,3				
Kardial	33	16	48,5				
Ophthalmologisch	33	9	27,3				
*Neurootologische Symptome*							
Vestibulärer Schwindel	33	13	39,4	–			
Hörminderung	33	14	42,4				
Tinnitus	33	14	42,4				
Sensorineurale Schwerhörigkeit	33	18	54,5				
Hörminderung bei Patienten < 45 Jahren	21	10	47,6				

*STD* Standardabweichung, *MAX* Maximum, *MIN* Minimum

Systemische Manifestationen umfassten in absteigender Reihenfolge Akroparästhesien (20/33), Manifestationen des Herzens (16/33), Apoplex (13/33), gastrointestinale Manifestationen (13/33) sowie renale Manifestationen, Angiokeratome und Augenmanifestationen.

Zu den HNO-Manifestationen bei Erstvorstellung gehörten ein subjektiver ein- oder beidseitiger Hörverlust (14/33), vestibulärer Schwindel (13/33) und/oder Tinnitus (14/33). Zudem stellten sich 36,4 % ohne jegliche HNO-Symptome vor. 63,6 % hatten mindestens ein Symptom. 12,1 % aller Patienten litten unter allen drei Symptomen. 14 Patienten beklagten sich über einen subjektiven Hörverlust. Ein relevantes neurootologisches Defizit wurde definiert als ein pathologisches Ergebnis in der BERA, im VOG oder als ein relevanter Hörverlust (nach dem 3‑Frequenz-Modell von Röser 1980). Mittels des Log-Rang-Test wurde der mittlere Zeitpunkt von der Erstdiagnose bis zum Auftreten eines relevanten neurootologischen Defizits mit 94,7 Monaten angegeben (Tab. 2).

**Table 2:** 

Einflussgröße		Gesamtzahl	Anzahl Ereignisse	Mittlere Zeit bis Ereignis	*p*-Wert
Alter bei Erstdiagnose (ED)	–	33	20	124,5	*p* = 0,117^(b)^
Geschlecht	w	17	6	110,7	*p* = 0,183^(a)^
	m	16	14	95,4	–
Akroparästhesien	Nein	13	5	65,6	*p* = 0,510^(a)^
	Ja	20	15	134,7	–
Hautveränderungen/Angiokeratome	Nein	23	11	152,8	*p* = 0,625^(a)^
	Ja	10	9	96,9	–
Neurologisch/Apoplex	Nein	20	10	94,2	*p* = 0,339^(a)^
	Ja	13	10	155,1	–
Gastrointestinal	Nein	20	12	124,8	*p* = 0,981^(a)^
	Ja	13	8	85,7	–
Nieren	Nein	22	13	82,8	*p* = 0,262^(a)^
	Ja	11	7	197,1	–
Herz	Nein	17	8	114,6	*p* = 0,983^(a)^
	Ja	16	12	136,6	–
Auge	Nein	24	13	125,2	*p* = 0,638^(a)^
	Ja	9	7	100,4	–
Enzymersatztherapie	Nein	9	3	38,9	*p* = 0,250^(a)^
	Ja	24	17	131,3	–

^(a)^ Log-Rang-Test

^(b)^ Cox-Regression

Ein im Audiogramm bestätigter sensorineuraler Hörverlust von > 25 dB HL bestätigte sich bei 18/33 Patienten (m = 12, w = 6). Das Durchschnittsalter aller Patienten mit einer Hörminderung lag insgesamt bei 44,72 (±11,4) Jahren. Bei nur 42,32 % der Patienten mit einer subjektiven Hörminderung zeigten somit 54,54 % eine sensorineurale Schwerhörigkeit von > 25 dB HL. Von den 18/33 Patienten hatten 16/33 einen beidseitigen Hörverlust und 2/33 einen einseitigen. Davon gaben 6 Patienten subjektiv keine Hörminderung an, die sich erst im Audiogramm bestätigte, wohingegen sich die subjektive Hörminderung eines Patienten im Audiogramm nicht bestätigen ließ.

21/33 der Patienten waren unter 45 Jahre. Davon hatten bereits 10 Patienten (m = 8 w = 2) einen ein- oder beidseitigen mittleren sensorineuralen Hörverlust von 41 dB HL (±29,64) bei 6 kHz bis 54 dB HL (±23,01) bei 8 kHz rechts und 36 dB HL (±12) bei 6 kHz bis 40 dB HL (±18,2) bei 8 kHz links. Zwei der Patienten hatten einen pantonalen rechtsseitigen plötzlichen Hörverlust von bis zu 90 dB HL.

**Figure 1:**
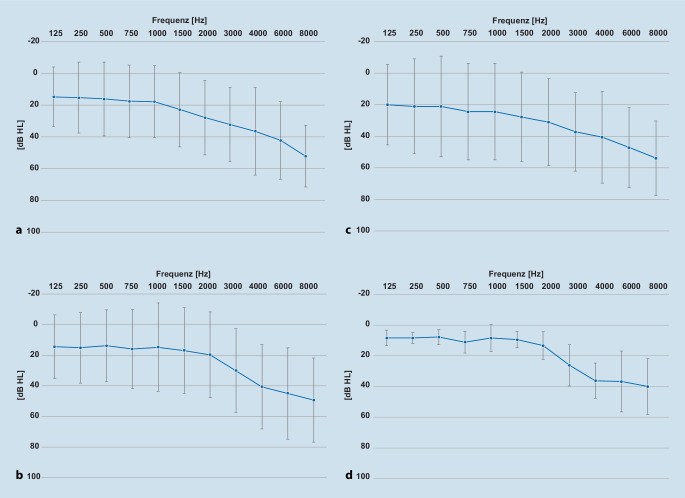

Die mittlere Hörschwelle aller Patienten (*n* = 18) mit einer Hörminderung von > 25 dB HL ist in den Abb. 1a rechts und 1b links dargestellt. Die Abb. 1c rechts und 1d links zeigt die mittlere Hörschwelle der Patienten unter 45 Jahren, bei denen sich eine sensorineurale Hörminderung zeigte.

Aus unserem Kollektiv konnten wir keine signifikante Altersabhängigkeit feststellen (t-Test für unabhängige Stichproben, *p* = 0,142). Es zeigte sich jedoch eine Signifikanz bezüglich einer relativen Hörminderung und des männlichen Geschlechts (*p* = 0,024) mittels Pearson-Chi-Quadrat-Test.

Die Tubenfunktion war bei allen Patienten normal, bestätigt durch ein normales Tympanogramm vom Typ A. Drei Patienten hatte ein pathologisches Ergebnis in der BERA.

In allen drei Fällen war die Jewett-I-Komponente nicht bestimmbar, und die Jewett-V-Komponente war verlängert. Eine retrocochleäre Störung wurde hierbei mittels MRT ausgeschlossen.

14 Patienten klagten über Tinnitus. 15 Patienten klagten über subjektiven Schwindel, 9 davon in Übereinstimmung mit pathologischen Ergebnissen im VOG. Bei vier Patienten war das VOG trotz des subjektiven Schwindels normal. Zwei Patienten mit Schwindel hatten zentrale Zeichen im VOG, verbunden mit neurologischen Symptomen.

Wir konnten eine signifikante Korrelation zwischen dem Vorhandensein von mindestens einem Ohrereignis und dem Auftreten oder der Zeit bis zum Auftreten eines weiteren neurootologischen Ereignisses erkennen (Log-Rang-Test, *p* = 0,018; Tab. 2).

Bei nachfolgenden Berechnungen mittels Kontingenztafelanalysen (Chi-Quadrat-Test) konnte ein signifikanter Zusammenhang zwischen dem Auftreten neurologischer Symptome (Apoplex) und einer Hörminderung (*p* = 0,001) sowie zwischen Herzmanifestationen und einer Hörminderung (*p* = 0,024) gezeigt werden (Tab. 3).

**Table 3:** 

Relative Hörminderung * Neurologisch/Apoplex												
*Kreuztabelle*						*Chi-Quadrat-Tests*						
–			*Neurologisch/Apoplex*		*Gesamt*	*–*	*Wert*	*Df*	*Asymptotische Signifikanz (2-seitig)*	*Exakte Signifikanz (2-seitig)*	*Exakte Signifikanz (einseitig)*	*Punktwahrscheinlichkeit*
			*Nein*	*Ja*								
Relative Hörminderung	Nein	Anzahl	15	2	17	*Pearson-Chi-Quadrat-Test*	11,211^a^	1	**0,001**	0,001	0,001	–
		% innerhalb Neurologisch/Apoplex	75,0 %	15,4 %	51,5 %	Kontinuitätsberichtigung^b^	8,951	1	*0,003*	–		
	Ja	Anzahl	5	11	16	Wahrscheinlichkeitsquotient	12,062	1	*0,001*	0,001	0,001	–
		% innerhalb Neurologisch/Apoplex	25,0 %	84,6 %	48,5 %	Exakter Test nach Fisher	–			0,001	0,001	
Total		Anzahl	20	13	33	Lineare Zuordnung	10,871^c^	1	*0,001*	0,001	0,001	0,001
		% innerhalb Neurologisch/Apoplex	100,0 %	100,0 %	100,0 %	Anzahl *n* an validen Fällen	33	–				
Relative Hörminderung * Herz												
*Kreuztabelle*						*Chi-Quadrat-Tests*						
–			*Herz*		*Total*	*–*	*Wert*	*Df*	*Asymptotische Signifikanz (2-seitig)*	*Exakte Signifikanz (2-seitig)*	*Exakte Signifikanz (einseitig)*	*Punktwahrscheinlichkeit*
			*Nein*	*Ja*								
Relative Hörminderung	Nein	Anzahl	12	5	17	*Pearson-Chi-Quadrat-Test*	5,107^d^	1	**0,024**	0,038	0,027	–
		% innerhalb Herz	70,6 %	31,3 %	51,5 %	Kontinuitätsberichtigung^e^	3,653	1	*0,056*	–		
	Ja	Anzahl	5	11	16	Wahrscheinlichkeitsquotient	5,246	1	*0,022*	0,038	0,027	–
		% innerhalb Herz	29,4 %	68,8 %	48,5 %	Exakter Test nach Fisher	–			0,038	0,027	
Gesamt		Anzahl	17	16	33	Lineare Zuordnung	4,952^f^	1	*0,026*	0,038	0,027	0,023
		% innerhalb Herz	100,0 %	100,0 %	100,0 %	Anzahl *n *an validen Fällen	33	–				

^a^ 0 Zellen (0,0 %) weisen eine erwartete Anzahl unter 5 auf. Die minimale erwartete Anzahl beträgt 6,30

^b^ Berechnet lediglich für eine 2 × 2-Tabelle

^c^ Die standardisierte Statistik lautet 3,297

^d^ 0 Zellen (0,0 %) weisen eine erwartete Anzahl unter 5 auf. Die minimale erwartete Anzahl beträgt 7,76

^e^ Berechnet lediglich für eine 2 × 2-Tabelle

^f^ Die standardisierte Statistik lautet 2,225

## Diskussion

Morbus Fabry (Fabry-Krankheit, FD) ist eine X‑chromosomal vererbte Störung des Glykosphingolipid-Stoffwechsels, die auf eine mangelhafte Aktivität des lysosomalen Enzyms α‑Galaktosidase A zurückzuführen ist [2].

Neben der fortschreitenden multilokulären Anhäufung von Glykosphingolipiden im Plasma und in den Lysosomen führt dies zu einer Multisystemstörung, die insbesondere das Nervensystem, die Haut, das Herz, die Nieren und die Augen betrifft. Zu den typischen Anfangssymptomen gehören Akroparästhesie, Hypohydrose, Angiokeratome der Haut und Hornhautdystrophie. In späteren Stadien führt die fortschreitende Ablagerung von Glykolipiden in den Endothel- und glatten Muskelzellen der Mikrogefäße zu einer Verengung und Thrombose der kleinen Arterien. Dies führt zu ischämischen Komplikationen mit Beteiligung des Gehirns und des Herzens in Verbindung mit einer chronischen Niereninsuffizienz und häufig frühem Tod [3, 9].

Das Auftreten von neurootologischen Symptomen im Rahmen von FD wird in der Literatur selten beschrieben. Jedoch kommt es bei Patienten mit Morbus Fabry häufiger als vermutet zu fortschreitendem Hörverlust und plötzlicher Ertaubung, meist in späteren Stadien der Krankheit [7, 9, 27]. Der Hörverlust zeigt sich meist in den hohen Frequenzen und scheint mit schweren Symptomen der Fabry-Krankheit wie Nierenversagen und zerebrovaskulären Läsionen zu korrelieren [9, 17]. Zudem wird ein Zusammenhang zwischen Schwerhörigkeit und Akroparästhesien sowie erhöhter Mikroalbuminurie vermutet [22].

In diese retrospektive Studie konnten 33 Patienten eingeschlossen werden, die sich routinemäßig (unabhängig vom Auftreten neurootologischer Symptome) mit bestätigtem Morbus Fabry in unserer HNO-Abteilung vorgestellt haben. Das Durchschnittsalter von 40,45 (±11,71) Jahren war relativ niedrig. Dies lässt sich auf den routinemäßigen Einschluss aller FD-Patienten zurückführen, somit auch Patienten mit der Neudiagnose eines FD ohne jegliche Symptomatik. In unserer Studie zeigte sich eine mittlere Inzidenz für einen fortschreitenden Hörverlust, plötzliche Taubheit und Tinnitus aurium. 14/33 (42,42 %) klagten über einen fortschreitenden oder plötzlichen subjektiven Hörverlust. Bestätigt wurde die sensorineurale Schwerhörigkeit von > 25 dB HL sogar bei 54,54 % der Patienten. 14/33 (42,42 %) Patienten hatten einen Tinnitus aurium, und 13/33 (39,39 %) Patienten klagten über ständigen oder intermittierenden Schwindel. In der Literatur wird die Prävalenz für einen subjektiven Hörverlust bei Fabry-Patienten mit 32,4 % und für Tinnitus mit 41,2 % angegeben. Auch hier war der Prozentsatz für einen bestätigten sensorineuralen Hörverlust mit 59 % höher und ähnlich zu unseren Ergebnissen [15, 16]. In der zitierten Literatur liegt das Durchschnittsalter jedoch um 5 Jahre höher als in unserer Studie.

Wenn man von einer Prävalenz von 30 % für eine Presbyakusis in der Allgemeinbevölkerung bei den 50- bis 60-jährigen Patienten ausgeht, kann man bei den älteren Patienten in unserem Kollektiv nicht alles auf die Fabry-Krankheit zurückführen [18].

Jedoch zeigen Völter et al. in einer Studie von 2021 einen durchschnittlichen Abfall im Hochtonbereich von 25 dB HL (6 Hz) bis 30 dB HL (8 kHz) bei 60-jährigen Männern auf 42 dB HL (6 kHz) bis 50 dB HL (8 kHz) bei 70-jährigen Männern [26]. In unserem Kollektiv liegt der Altersdurchschnitt aller Patienten mit einem sensorineuralem Hörverlust bei 44,7 (±11,3) Jahren, also deutlich jünger, und der mittlere Hörverlust liegt bei 42,2 dB HL (±24,4) bei 6 kHz und 52,2 dB HL (19,4) bei 8 kHZ und somit höher als der Durchschnitt, was den Zusammenhang mit FD erklären kann.

21/33 der Patienten waren unter 45 Jahre. Davon hatten bereits 10 Patienten einen sensorineuralen Hörverlust. Morbus Fabry ist eine seltene Erkrankung, sollte aber bei plötzlichem Hörverlust und gegebenenfalls familiärer Häufung dennoch als Differenzialdiagnose in Betracht gezogen und getestet werden.

Die Signifikanz bezüglich einer relativen Hörminderung und des männlichen Geschlechts ist bei der Fallzahl und Verteilung von Männern und Frauen nur bedingt aussagekräftig, zeigt aber eine klare Tendenz und ist in Übereinstimmung mit der Literatur [4, 9]. Da der klassische Phänotyp von FD bei dieser x‑chromosomalen Erkrankung bei männlichen Patienten zu finden ist, erklärt dies den höheren Anteil an männlichen Patienten. Die nicht klassische Varianten der FD treten häufiger als die klassischen Phänotypen auf [1]. Das Auftreten der Symptome wird später beschrieben und ist in der Regel auf Herz und Niere beschränkt. Diese niedrigen Inzidenzen in unserer Kohorte könnten darauf zurückzuführen sein, dass auch Patienten ohne neurootologische Symptome routinemäßig vorgestellt wurden. Da in vielen Fällen die neurootologischen Symptome erst in einem späteren Stadium der Erkrankung auftreten, könnte der Zeitpunkt des Auftretens auch nach der Vorstellung bei uns liegen. Unter Berücksichtigung des Alters war der jüngste Patient mit neurootologischen Symptomen 20 Jahre alt, der älteste Patient war 58 Jahre alt. Unsere Kohorte setzte sich also aus einem breiten Altersspektrum an Patienten zusammen, wobei auch junge Patienten mit neurootologischen Symptomen vorstellig wurden. Alle Patienten in unserer Kohorte hatten eine normale Tubenfunktion mit einem Tympanogramm vom Typ A und keinerlei Schallleitungskomponente.

Männer scheinen bei fast allen Symptomen der Fabry-Krankheit stärker betroffen zu sein. Dies ist wahrscheinlich auf eine höhere Aktivität des Enzyms α‑Galaktosidase A bei Frauen zurückzuführen, da sie noch ein „gesundes“ X‑Chromosom besitzen [20]. Der vor allem die höheren Frequenzen betreffende Hörverlust, der auch in unserer Kohorte festgestellt wurde, scheint überwiegend auf einen Verlust von Haarzellen in der basalen Cochlea zurückzuführen zu sein [12, 23]. Bei einer signifikanten Korrelation mit neurologischen sowie kardiovaskulären Manifestationen wäre prinzipiell auch ein vaskulärer Verschluss der Arteria cochlearis denkbar.

Eine Korrelation zwischen einem plötzlichen oder fortschreitenden Hörverlust und vestibulärem Schwindel wurde bisher nicht beschrieben. Da in der Literatur nur selten vestibuläre Symptome bei Fabry-Patienten beschrieben sind, untersuchten wir auch die Koinzidenz von Hörverlust und vestibulären Symptomen anhand der Anamnese, der Untersuchung mittels Frenzel-Brille und der kalorischen Prüfung der Gleichgewichtsorgane mittels Videookulographie. Conti und Sergi stellten bei 29 % ihrer Patienten Anomalien in der vestibulären Diagnostik fest [4]. Damit ist unsere Inzidenz höher, wobei die vestibuläre Komponente nur teilweise objektivierbar war.

## Fazit für die Praxis

Eine Schallempfindungsschwerhörigkeit kommt bei Patienten mit Morbus Fabry häufig vor und ist nicht nur auf den klassischen Phänotyp bei Männern beschränkt. Eine engmaschige HNO-Kontrolle und ein Screening sollten vor allem bei kardiologischen und neurologischen Symptomen in Betracht gezogen werden, um frühe neurootologische Defizite zu erkennen und frühzeitig reagieren zu können. Vor allem bei jüngeren Patienten mit einem plötzlichen ein- oder beidseitigen Hörverlust und insbesondere bei einer familiären Häufung sollte ein Morbus Fabry in Betracht gezogen und getestet werden. Nach dem Auftreten von kardiologischen und/oder neurologischen Manifestationen ist das Auftreten neurootologischer Symptome wie Hörsturz, Schwindel oder Tinnitus im Verlauf der Erkrankung wahrscheinlich. Hierbei sind Männer häufiger betroffen als Frauen.
